# Conductivity image enhancement in MREIT using adaptively weighted spatial averaging filter

**DOI:** 10.1186/1475-925X-13-87

**Published:** 2014-06-26

**Authors:** Tong In Oh, Hyung Joong Kim, Woo Chul Jeong, Hun Wi, Oh In Kwon, Eung Je Woo

**Affiliations:** 1 Department of Biomedical Engineering and Impedance Imaging Research Center, Kyung Hee University, 446-701 Yongin, Korea; 2 Department of Mathematics, Konkuk University, 143-701 Seoul, Korea

**Keywords:** Magnetic resonance electrical impedance tomography, Conductivity image, Noise estimation, Denoising method, Adaptively weighted spatial averaging filter

## Abstract

**Background:**

In magnetic resonance electrical impedance tomography (MREIT), we reconstruct conductivity images using magnetic flux density data induced by externally injected currents. Since we extract magnetic flux density data from acquired MR phase images, the amount of measurement noise increases in regions of weak MR signals. Especially for local regions of MR signal void, there may occur excessive amounts of noise to deteriorate the quality of reconstructed conductivity images. In this paper, we propose a new conductivity image enhancement method as a postprocessing technique to improve the image quality.

**Methods:**

Within a magnetic flux density image, the amount of noise varies depending on the position-dependent MR signal intensity. Using the MR magnitude image which is always available in MREIT, we estimate noise levels of measured magnetic flux density data in local regions. Based on the noise estimates, we adjust the window size and weights of a spatial averaging filter, which is applied to reconstructed conductivity images. Without relying on a partial differential equation, the new method is fast and can be easily implemented.

**Results:**

Applying the novel conductivity image enhancement method to experimental data, we could improve the image quality to better distinguish local regions with different conductivity contrasts. From phantom experiments, the estimated conductivity values had 80% less variations inside regions of homogeneous objects. Reconstructed conductivity images from upper and lower abdominal regions of animals showed much less artifacts in local regions of weak MR signals.

**Conclusion:**

We developed the fast and simple method to enhance the conductivity image quality by adaptively adjusting the weights and window size of the spatial averaging filter using MR magnitude images. Since the new method is implemented as a postprocessing step, we suggest adopting it without or with other preprocessing methods for application studies where conductivity contrast is of primary concern.

## Background

Electrical conductivity is a passive material property of a biological tissue or organ providing diagnostic information of its physiological function and pathological state [1,2]. In magnetic resonance electrical impedance tomography (MREIT), we aim to visualize the internal conductivity distribution of the human body by injecting electrical current and measuring induced magnetic flux density data using an MRI scanner [3,4]. The MREIT technique uses the *z*-component *B*_*z*_ of the magnetic flux density **B**=(*B*_*x*_,*B*_*y*_,*B*_*z*_) to recover conductivity and/or current density images [5-10].

In experimental MREIT studies of animal and human subjects [11-14], the quality of reconstructed conductivity images highly depends on the noise level in measured *B*_*z*_ data and the injection current amplitude. For a given current amplitude, we can improve the image quality by reducing the noise level. If we reduce the current amplitude to avoid adverse effects such as electrical stimulations of nerve and muscle, the range of *B*_*z*_ decreases proportionally and the measured *B*_*z*_ data become more vulnerable to noise. In order not to deteriorate the conductivity image quality, we should reduce the noise level as well.

There have been numerous studies to minimize the noise level in measured *B*_*z*_ data by optimizing the data collection method including pulse sequences, RF coils, shimming, averaging, and so on. For example, the injected current nonlinear encoding (ICNE) method was introduced to reduce the noise level in *B*_*z*_ data by extending the current injection time without increasing the scan time [15]. Various multi-echo and multi-coil techniques have been developed together with optimization methods to combine multiple signals from multiple coils and echoes [16].

Once we acquire *k*-space data from a designed MREIT experiment, we should carefully process the data to suppress the noise and enhance the image quality. This requires proper understanding of the noise characteristics. The noise standard deviation of *B*_*z*_ is inversely proportional to the signal-to-noise ratio (SNR) of the MR magnitude image and the current injection time when the magnitude image SNR is higher than about 3 [17,18]. Since the magnitude image SNR varies over the image, the noise level of *B*_*z*_ also varies. If the magnitude image SNR is much lower, for example, less than 3, then we should expect an excessive amount of noise in *B*_*z*_. This may happen when we scan an animal or human subject since there exist local regions of weak MR signal such as the lungs, gas-filled organs, and outer layers of bones. In those regions with very low magnitude image SNRs, there occur excessive amounts of noise in measured *B*_*z*_ data. It is, therefore, important to properly suppress the noise effects in preprocessing or postprocessing steps.

There are several denoising methods to process the acquired *B*_*z*_ data [19-23]. These methods use either a PDE-based approach or a localized data processing approach and require a certain degree of manual adjustment of parameters. All of these methods are applied to measured *B*_*z*_ data before conductivity image reconstructions as a preprocessing step.

In this paper, we present a new method to suppress the noise effects in reconstructed conductivity images as a postprocessing step. We suggest incorporating *a prior* information from the MR magnitude image which is always available in MREIT. For current density imaging using measured magnetic flux density data, Joy *et al*. suppressed noise effects by setting some appropriate threshold in the MR magnitude image SNR [24]. A similar approach based on the morphological enabled dipole inversion (MEDI) has been proposed in quantitative susceptibility map (QSM) to overcome the ill-posedness in its inverse problem [25].

The proposed method enhances the quality of reconstructed conductivity images by using an adaptively weighted spatial averaging filter. To determine the weights, we will define a distance function with respect to the noise standard deviation in measured *B*_*z*_ data. Since the noise is inversely proportional to the MR magnitude image, we will incorporate the magnitude image in the spatial filtering process. After describing the details of the proposed method, we will show how it performs with experimental data from a conductivity phantom and also animal subjects.

## Methods

### Noise in magnetic flux density data

We inject current into an imaging object, of which timing is synchronized with a chosen MR pulse sequence. The usual current injection period is from the end of the exciting RF pulse to the beginning of the readout gradient. One may choose different current injection times and patterns depending on designed pulse sequences. For example, the ICNE-multi-echo pulse sequence extends the duration of current injection until the end of multiple read-out gradients.

The externally injected current produces an internal magnetic flux density distribution and its *z*-component *B*_*z*_ in a voxel results in an extra phase in the MR phase image. To remove systematic phase artifacts, we sequentially inject positive *I*^+^ and negative *I*^-^ current pulses to obtain the following *k*-space data:

(1) $$S^{\pm }\left(k_{x},,,k_{y}\right)=\int_{\Omega }M\left(x,,,y\right)e^{\mathrm{i\delta}\left(x,,,y\right)}e^{\pm \mathrm{i\gamma}B_{z}\left(x,,,y\right)T_{c}}e^{i2\pi \left(k_{x},x,+,k_{y},y\right)}\text{dxdy}$$

where *M* is the MR magnitude image, *δ* is any systematic phase artifact, *γ*=26.75×10^7^rad/T·s is the gyromagnetic ratio of the proton, and *Ω* is a field-of-view (FOV). Here, the superscript of *S*^±^ denotes a brief notation for *S*^+^ and *S*^-^.

We extract the magnetic flux density *B*_*z*_ by

(2) $$B_{z}\left(x,,,y\right)=\frac{1}{2\gamma T_{c}}\;\text{arg}\;\left(\frac{\Gamma^{+}\left(x,,,y\right)}{\Gamma^{-}\left(x,,,y\right)}\right)$$

where $\Gamma^{\pm }=Me^{\mathrm{i\delta}}e^{\pm \mathrm{i\gamma}B_{z}T_{c}}$.

The noise standard deviation of the measured magnetic flux density *B*_*z*_ is inversely proportional to the current injection time *T*_*c*_ and the SNR of the MR magnitude image *Υ* as

(3) $${\text{sd}}_{B_{z}}=\frac{1}{2\gamma T_{c}\Upsilon }$$

for *Υ*>2.8 [17,18]. If we reduce the current amplitude *I*^±^, the range of *B*_*z*_ decreases proportionally. To reduce the noise standard deviation, we have to increase the current injection time *T*_*c*_ and the SNR *Υ* simultaneously. However, this is not possible mainly due to the *T*_2_ or $T_{2}^{*}$ decay of the MR signal.

### Conductivity image reconstruction

The externally injected current *I* induces distributions of voltage *u*, current density **J**=(*J*_*x*_,*J*_*y*_,*J*_*z*_), and magnetic flux density **B**=(*B*_*x*_,*B*_*y*_,*B*_*z*_) inside the imaging object *Ω* with its boundary *∂Ω*. The voltage satisfies the following partial differential equation:

(4) $$\left\{\begin{array}{cccc} \nabla \cdot \left(\sigma ,\left(r\right),\nabla ,u,\left(r\right)\right) & = & 0\;\; & \text{in}\;\Omega \\ \;-\sigma \nabla u\cdot n & = & g\;\; & \text{on}\;\partial \Omega \end{array}\right)$$

where **r**=(*x*,*y*,*z*), **n** is the outward unit normal vector on *∂Ω*, and *g* denotes the Neumann boundary data subject to the injection current. From the Biot-Savart law, the magnetic flux density *B*_*z*_ is related with the current density **J**=-*σ*∇*u* as

(5) $$B_{z}\left(r\right)=\frac{\mu_{0}}{4\pi }\int_{\Omega }\frac{\left(y,-,y^{\prime }\right)J_{x}\left(r^{\prime }\right)-\left(x,-,x^{\prime }\right)J_{y}\left(r^{\prime }\right)}{|r-r^{\prime }{|}^{3}}dr^{\prime }$$

where *μ*_0_ is the magnetic permeability of the free space.

There exists numerous image reconstruction algorithms to visualize the conductivity *σ* or the current density **J** in the imaging object from measured *B*_*z*_ data [5,9,16,26]. In this paper, we adopted the transversal *J*-substitution algorithm [27] since it differentiates the noisy *B*_*z*_ data once and does not propagate the noise effects from one region to another. We first assume the imaging object with a homogeneous conductivity distribution *σ*^*H*^. Solving (4) with *σ*^*H*^ in place of *σ*, we can compute the voltage, current density, and magnetic flux density, which are denoted as *u*^*H*^, **J**^*H*^, and $B_{z}^{H}$, respectively.

In MREIT experiments, we measure two *B*_*z*_ data subject to two orthogonal injection currents. Denoting them as *B*_*z*,1_ and *B*_*z*,2_, the transversal *J*-substitution algorithm produces an image of the conductivity *σ* as

(6) $$\begin{array}{c} \sigma \left(r\right)=\sigma^{H}\left(r\right)-\frac{1}{\mu_{0}}\frac{\overset{2}{\underset{n=1}{\sum }}{\tilde{\nabla }}^{⊥}\left(B_{z,n}\left(r\right)-B_{z,n}^{H}\left(r\right)\right)\cdot \tilde{\nabla }u_{n}^{H}\left(r\right)}{\overset{2}{\underset{n=1}{\sum }}\langle \tilde{\nabla }u_{n}^{H}\left(r\right),\tilde{\nabla }u_{n}^{H}\left(r\right)\rangle } \end{array}$$

where ${\tilde{\nabla }}^{⊥}f:=\left(\frac{\mathrm{\partial f}}{\mathrm{\partial y}},-\frac{\mathrm{\partial f}}{\mathrm{\partial x}}\right)$ for a given scalar function *f* and 〈·,·〉 denotes the scalar inner product. We used the computed values of two magnetic flux densities $B_{z,1}^{H}$ and $B_{z,2}^{H}$ corresponding to two computed voltages $u_{1}^{H}$ and $u_{2}^{H}$, respectively, for the homogeneous case of *σ*^*H*^. Note that the noisy data of *B*_*z*,1_ and *B*_*z*,2_ may deteriorate the quality of the reconstructed image of *σ*. More details about the image reconstruction algorithm are described in [27].

### Adaptively weighted spatial averaging

To determine a neighborhood of a pixel, we define the following distance function:

(7) $$D\left(r,,,s\right):=\frac{\left|M\left(r\right)-M\left(s\right)\right|}{h\left(r\right)},\;s\in B_{r}\left(\eta ,\left(r\right)\right)$$

where *M*(**r**) is the MR magnitude image, *B*_**r**_(*η*(**r**)) is a neighborhood of the point **r** with a radius *η*(**r**), and *h*(**r**) is a function of the noise level in measured *B*_*z*_ data. For each pixel **s** in the neighborhood of the pixel **r**, that is, **s**∈*B*_**r**_(*η*(**r**)), we define the following weighting factor *w*_**r**_(**s**):

(8) $$w_{r}\left(s\right):={\frac{1}{\zeta }}_{r}e^{-D\left(r,,,s\right)}$$

where $\zeta_{r}:=\underset{s}{\sum }e^{-D\left(r,,,s\right)}$ is a normalization constant ensuring that $\underset{s}{\sum }w_{r}\left(s\right)=1$. We perform the adaptive spatial averaging of the reconstructed conductivity values as the following weighted sum:

(9) $$\sigma_{w}\left(r\right)=\underset{s\in B_{r}\left(\eta ,\left(r\right)\right)}{\sum }w_{r}\left(s\right)\sigma \left(s\right).$$

For the pixel at **r**, the distance *D*(**r**,**s**) measures the similarity of *M*(**r**) and *M*(**s**) in the surrounding region of **r**. Since the noise standard deviation of *B*_*z*_ is inversely proportional to the magnitude image SNR, the weights are in effect adjusted by the noise level in measured *B*_*z*_ data. In (7), two parameters of *η*(**r**) and *h*(**r**) regulate the extent of the spatial averaging filter. We now design the denominator *h*(**r**) and the radius *η*(**r**) in (7) as

(10) $$h\left(r\right)\propto {\text{sd}}_{B_{z}}\left(r\right)\;\text{and}\;\eta \left(r\right)\propto {\text{sd}}_{B_{z}}\left(r\right).$$

We can choose the proportionality parameters of *h*(**r**) and *η*(**r**) in (10) depending on the quality of the reconstructed conductivity image to be filtered.

The designed distance function *D*(**r**,**s**) preserves the conductivity value where the noise level of *B*_*z*_ is low. To determine the characteristics of the weighting factor *w*_**r**_(**s**), we decompose the weighted conductivity *σ*_*w*_(**r**) to the true conductivity *σ*_*t*_(**r**) and the noise term:

(11) $$\sigma_{w}\left(r\right)=\underset{s\in B_{r}\left(\eta ,\left(r\right)\right)}{\sum }w_{r}\left(s\right)\sigma \left(s\right)=\underset{s\in B_{r}\left(\eta ,\left(r\right)\right)}{\sum }w_{r}\left(s\right)\left(\sigma_{t}\left(s\right)+N_{r}\left(s\right)\right)$$

where *σ*_*t*_(**s**) and *N*_**r**_(**s**) denote the noiseless true conductivity and the noise term at **s**, respectively. The weighted conductivity *σ*_*w*_(**r**) and the true conductivity *σ*_*t*_(**r**) satisfy the following relation:

(12) $$\begin{array}{l} \left|\sigma_{w}\left(r\right)-\sigma_{t}\left(r\right)\right|=\frac{1}{\zeta_{r}}\left|\underset{s\in B_{r}\left(\eta ,\left(r\right)\right)}{\sum }e^{-D\left(r,,,s\right)}\left(\sigma_{t}\left(s\right)-\sigma_{t}\left(r\right)+N_{r}\left(s\right)\right)\right|\\ \;\leq E_{1}\left(r\right)+E_{2}\left(r\right) \end{array}$$

where

(13) $$E_{1}\left(r\right)=\frac{1}{\zeta_{r}}\left|\underset{s\in B_{r}\left(\eta ,\left(r\right)\right)}{\sum }e^{-D\left(r,,,s\right)}\left(\sigma_{t},\left(s\right),-,\sigma_{t},\left(r\right)\right)\right|$$

and

(14) $$E_{2}\left(r\right)=\frac{1}{\zeta_{r}}\left|\underset{s\in B_{r}\left(\eta ,\left(r\right)\right)}{\sum }e^{-D\left(r,,,s\right)}N_{r}\left(s\right)\right|.$$

For a small amount of noise, both *h*(**r**) and *η*(**r**) are small and, therefore, the relation (12) implies that both $E_{1}\left(r\right)$ and $E_{2}\left(r\right)$ are small. This means that the method does no harm to the conductivity image when the noise level is low.

For a large amount of noise, the first error term $E_{1}\left(r\right)$ becomes small if there was no significant variations of the conductivity values within the neighboring region *B*_**r**_(*η*(**r**)) of the pixel at **r**. This is true only when the pixels with similar magnitude image values also have similar conductivity values. We will discuss implications of this restriction later. The second error term $E_{2}\left(r\right)$ is considerably reduced by the spatial averaging of the random noise.

### Imaging experiments

#### Phantom imaging

**Figure 1:**
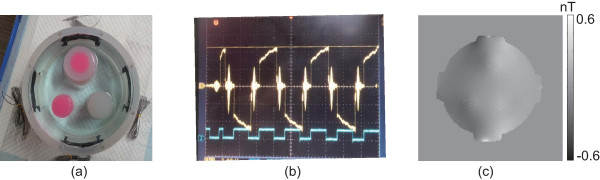
**Experimental set-up and measured magnetic flux density image. (a)** Phantom with three objects with different conductivity values. **(b)** Injection current pulses synchronized with RF pulses. **(c)** Measured magnetic flux density *B*_*z*_ image subject to vertical current injection.

To evaluate the performance of the proposed method, we scanned a cylindrical phantom with four carbon-hydrogel electrodes (HUREV Co. Ltd, Korea). The phantom was filled with 0.4 S/m saline and included three cylindrical objects. The first ($D_{1}$) was a TX-151 object (1.5 S/m), the second ($D_{2}$) was an agar object (0.1 S/m), and the third ($D_{3}$) was a TX-151 object (1.5 S/m) wrapped with an agar object (0.3 S/m) as shown in Figure 1(a). We placed the phantom inside the bore of our 3 T MRI scanner (Magnum 3, Medinus Co. Ltd., Korea). Using a custom-designed MREIT current source [28], we injected 10 mA currents in the horizontally and vertically directions. The parameters of the spin-echo-based imaging sequence were as follows: repetition time *T*_*R*_=1200 ms, echo time *T*_*E*_=15 ms, number of echoes *N*_*E*_=5, field of view (FOV) was 240×240 mm ^2^, number of excitations (NEX) was 1, imaging matrix was 128×128, and total imaging time was 10.24 min. Figure 1(b) shows the injected current pulses synchronized with RF pulses. We combined the acquired multiple echoes to optimize the SNR in measured *B*_*z*_ data [16]. Figure 1(c) shows the measured *B*_*z*_ image subject to the vertical current injection.

#### Animal imaging

**Figure 2:**
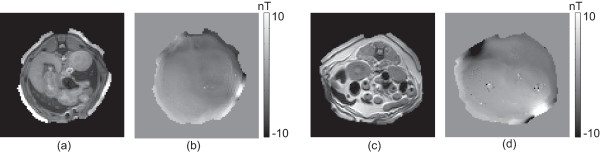
**MR magnitude and magnetic flux density images for animal experiments. (a)** and **(b)** are MR magnitude and *B*_*z*_ images, respectively, of the upper abdominal region of the first beagle. **(c)** and **(d)** are MR magnitude and *B*_*z*_ images, respectively, of the lower abdominal region of the second beagle.

The animals were laboratory beagles (2–3 years old, weighing 8–15 kg) without having any known disease. To prevent dribbling, we injected 0.1 mg/kg of atrophine sulfate. Ten minutes later, we anesthetized the dog with intramuscular injection of 0.2 ml/kg Tiletamine and Zolazepam (Zoletil 50, Virbac, France). Twenty minutes later, we sacrificed it with an intravenous injection of 80 mg/kg KCL (Entobar, Hanrim Pharmacy, Korea). After clipping the hair, we attached four carbon-hydrogel electrodes (HUREV Co. Ltd., Korea) around the imaging area. The size of each electrode was 80×80×6 mm ^3^. The procedure was approved by the Institutional Animal Care and Use Committee (IACUC). We injected currents in two mutually orthogonal directions between two pairs of electrodes facing each other. The injection current amplitude ranged from 5 to 10 mA. We adopted the ICNE pulse sequence. The imaging parameters were as follows: repetition time *T*_*R*_=1200 ms, echo time *T*_*E*_=30 ms, FOV was 280×280 mm ^2^, slice thickness was 4 mm, number of slice was 8, NEX was 6, imaging matrix was 128×128, and total imaging time was 60 min. Figure 2 shows two data sets we used in the paper.

## Results

**Figure 3:**
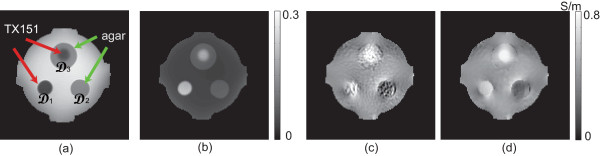
**Experiment results for the phantom. (a)** MR magnitude image of the phantom in Figure 1. **(b)** Computed values of the denominator *h* in (7). **(c)** Conductivity image without using any filtering. **(d)** Conductivity image after applying the adaptively weighted spatial averaging filter.

Figure 3(a) shows the MR magnitude image at the middle slice of the phantom. We segmented three regions of $D_{1}$ (TX-151), $D_{2}$ (agar), and $D_{3}$ (TX-151 wrapped with agar). Depending on the noise level in measured *B*_*z*_ data, we determined the denominator *h* in (7) to compute the distance function *D*(**r**,**s**). Figure 3(b) shows the denominator *h* in (7). Figure 3(c) shows the reconstructed conductivity image using the algorithm in (6). Due to the short *T*_2_ relaxation time of the objects, the reconstructed conductivity values of the objects show strong noise defects. Figure 3(d) shows the filtered conductivity image using the proposed method. In the noisy regions of $D_{i},\;i=1,2,3$, the values of the denominator *h* were relatively large to produce smaller distance values in (7). Since the values of *η* were also large in $D_{i}$, more pixels in the neighboring region were included in the spatial averaging with relatively larger weights.

We may quantify the effects of the filtering method by computing the amount of conductivity changes in a homogeneous region. We defined the following variance function to estimate how the reconstructed conductivity values varied in each region of interest (ROI):

(15) $$V_{D_{i}}\left(\sigma \right):=\frac{1}{\left|D_{i}\right|}{\left(\int_{D_{i}}{\left|\nabla \sigma \right|}^{2}dr\right)}^{1/2}\;\text{for}\;i=1,2,3$$

where $\left|D_{i}\right|$ denotes the volume of the ROI $D_{i}$. Table 1 shows that the proposed method significantly improved the image quality.

**Table 1:** **Variance of the reconstructed conductivity values in the ROIs using the variance function in (**15**)**

	$V_{D_{1}}\left(\sigma \right)$	$V_{D_{2}}\left(\sigma \right)$	$V_{D_{3}}\left(\sigma \right)$
Before filtering	25.4	15.7	18.4
After filtering	3.39	3.89	4.81

**Figure 4:**
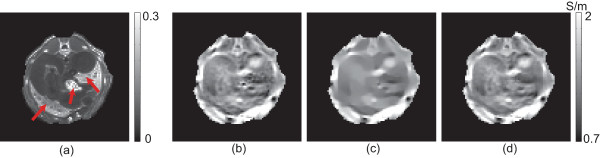
**Experiment results for the upper abdominal region. (a)** Computed values of the denominator *h* in (7) for the upper abdominal region in Figure 2(a). **(b)** Conductivity image without using any filtering. **(c)** Filtered conductivity image by the iteration method in (16). **(d)** Conductivity image after applying the adaptively weighted spatial averaging filter.

Figure 4(a) is the image of the denominator *h* in (7) for the upper abdominal region in Figure 2(a). The areas marked by the arrows have large values of *h* since their MR magnitude image SNRs were low. The average magnitude intensity in the marked areas was 3.78, where the intensity of the entire image ranged from 0 to 30. Since the noise level of *B*_*z*_ is inversely proportional to the MR magnitude intensity, the recovered conductivity values of the marked regions included a relatively large amount of noise. Figure 4(b) shows the reconstructed conductivity image without using any filtering method and we can observe severe noise effects in those regions.

To compare the proposed method with a conventional denoising technique, we used the following reaction-diffusion iteration step [29]:

(16) $$\left\{\begin{array}{l} \;v^{n+1}\left(x,,,y\right)=v^{n}\left(x,,,y\right)+\alpha \tilde{\nabla }\cdot \left(\frac{\tilde{\nabla }v^{n}}{\left|\tilde{\nabla }v^{n}\right|}\right)-\beta \left(v^{n},-,f\right)\\ \;v^{0}\left(x,,,y\right)\;=\;f\left(x,,,y\right) \end{array}\right)$$

where *f* is the reconstructed conductivity image shown in Figure 4(b). Figure 4(c) shows the denoised conductivity image using the iteration method (16). We can see that the iteration method blurred the entire image. Figure 4(d) is the conductivity image after applying the adaptively weighted spatial averaging filter. We can see that the proposed method enhanced the conductivity image in the marked regions without affecting other regions. For the blurred image in Figure 4(c), we used the following parameters: iteration number = 200, *α*=0.1, and fidelity term *β*=0.01. These parameters were chosen to make the variance of the conductivity values in the marked regions of the image in Figure 4(c) to be equal to that of the image in 4(d).

**Figure 5:**
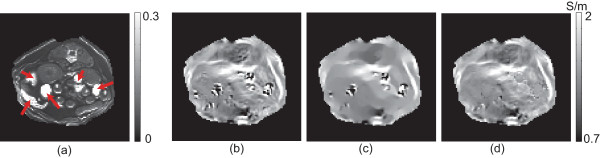
**Experiment results for the lower abdominal region. (a)** Computed values of the denominator *h* in (7) for the lower abdominal region in Figure 2(c) and (d). **(b)** Conductivity image without using the filtering. **(c)** Filtered conductivity image by the iteration method in (16). **(d)** Conductivity image after applying the adaptively weighted spatial averaging filter.

Figure 5 shows similar results for the lower abdominal region shown in Figure 2(c). The average magnitude intensity of the marked regions was 1.97 for this case and the measured *B*_*z*_ data were noisier than the case in Figure 4. Comparing two filtered conductivity images in 5(c) and (d), we can see that the method proposed in this paper is clearly advantageous.

## Discussion and conclusion

For animal or human subjects, their internal structures are heterogenous and there often exist local regions of weak MR signals. This makes the noise level in measured magnetic flux density data vary significantly for different pixels. Since we use the data to reconstruct conductivity images, noise effects are conveyed to the conductivity images with spatially varying image quality. Conventional filtering methods without considering this property, therefore, unnecessarily blur the entire conductivity image.

In this paper, we proposed a fast and simple method to enhance the conductivity image quality by utilizing the MR magnitude image. Noting that the noise level in measured magnetic flux density data is inversely proportional to the pixel value of the MR magnitude image, we could adaptively adjust the weights and window size of the spatial averaging filter. In designing the filter, we used two parameters of *η*(**r**) and *h*(**r**) in (7) to regulate the extent of the spatial averaging filter. Since we set their values to be proportional to the noise level in measured *B*_*z*_ data, the proposed method adaptively changes the extent of the spatial averaging depending on the noise level at each pixel. It is important to properly choose the proportionality constants for *η*(**r**) and *h*(**r**) in (10). Though we heuristically chose the constants in this paper, we plan to rigorously investigate their effects on the filtered image and develop an automatic method to determine them.

Since the spatial averaging is performed on neighboring pixels with similar MR magnitude image values, it may remove any useful conductivity contrast among those pixels. Using the proposed method, we can apply the spatial averaging to local regions of severe noise where we can not trust reconstructed conductivity values. We can, therefore, suppress noise for the price of reduced image resolution within local regions with large amounts of noise. The proposed method negligibly influences the conductivity image in other regions with enough SNR.For the phantom image in Figure 3, we found that the spatial averaging recovered the correct conductivity values in the noisy regions since there was no conductivity contrast within each region. For the animal images in Figures 4 and 5, it was difficult to assert that the recovered conductivity values of the noisy regions were correct since there could have been some conductivity contrast among those pixels with similar MR magnitude image values. In application studies, we may analyze both unfiltered and filtered conductivity images if quantitative conductivity values of noisy regions are of primary concern.

While the existing denoising methods in MREIT are applied to measured *B*_*z*_ data before conductivity image reconstructions [19-23], the new method proposed in this paper is a postprocessing method, which can be applied to reconstructed conductivity images. Without requiring any image segmentation, one can easily implement the method without or with chosen preprocessing steps. We suggest adopting the proposed method in future experimental MREIT studies where conductivity contrast is of primary concern to extract diagnostic information.

## Competing interests

The authors declare that they have no competing interests.

## Authors’ contributions

TIO and OIK developed the algorithm and wrote the manuscript. WCJ and HW prepared and performed the experiments and wrote up the experimental section. HJK and EJW conceived the idea and gave critical revision for important intellectual content. OIK designed the research topic and experiments, analyzed and drafted the manuscript. All authors read and approved the final manuscript.
